# Supplementary material for: Modulation of the tick gut milieu by a secreted tick protein favors Borrelia burgdorferi colonization
Source: Nat Commun. 2017 Aug 4;8:184. doi: 10.1038/s41467-017-00208-0 (PMC5543126; doi:10.1038/s41467-017-00208-0)
Supplement: Supplementary file 1 — Supplementary Information [file 41467_2017_208_MOESM1_ESM.pdf]

File name: Supplementary Information
Description: Supplementary Tables and Supplementary Figures

File name: Peer Review File
Description:

Accession number	Protein Encoded	Function/domains
ISCW022329	Transmembrane protein (Steroidogenic Acute Regulatory STAR)	Possible role in lipid transfer
ISCW009548	Hypothetical secreted protein	Unknown
ISCW019584	Phospho-lipid-hydroperxode Glutathione peroxidase	Reduction of lipid and hydroperoxides
ISCW016022*	Cytochrome C oxidase subunit I	Heme-Cu oxidase I superfamily, key enzyme in proton pump coupled to ATP generation [7]
ISCW007844	Short chain alcohol dehydrogenase	Role in metabolism of lipids, amino acids, carbohydrates and hormones [8]
ISCW020757*	Cytochrome C Oxidase III	Key enzyme in proton pump coupled to ATP generation
ISCW004817	Similar to Exocyst complex component 6B	Vesicular traffic of proteins from the golgi apparatus
ISCW003205	Hypothetical protein	Unknown
ISCW005926	Scapularisin-5	Defensin_2 superfamily, Antibacterial defense [18]
ISCW011165	3-Oxoacyl [acyl-carrier-protein] reductase	Fatty acid and polyunsaturated fatty acid biosynthesis
ISCW012492	Hypothetical protein	Unknown
ISCW019584	Phospholipid-hydroperoxide glutathione peroxidase	Glutathione metabolism-quenching of hydroperoxide
ISCW018900	Hypothetical protein	Unknown
ISCW014515	Secreted salivary protein	Unknown
ISCW015983	PeptidaseC13 family/aspariginyl peptidase	Bloodmeal digestion [10]
ISCW012432	Short chain alcohol dehydrogenase	Role in metabolism of lipids, amino acids, carbohydrates and hormones [8]

ISCW003426	Glutathione S-Transferase (Thioredoxin superfamily)	Detoxification of xenobiotic and endobiotic compounds [29]
ISCW012239	Secreted salivary protein -Salp9pac	Inhibition of host complement by binding MBL [12]
**KY629420**	Secreted protein with a Reeler domain	Defense against bacterial and fungal pathogens [19]
ISCW018909	Putative secreted protein	Unknown
ISCW003983	Oxysterol binding protein	Lipid transport and signaling [13]
ISCW007199	Villin/gelsolin domain-containing protein	Actin binding and regulation of actin assembly [21]
ISCW007338	Erlin-2 (Endoplasmic reticulum lipid raft protein), putative protein	Lipid raft associated protein
ISCW017045	Conserved hypothetical protein (Catenin-beta-like ARM protein superfamily)	Cell-cell communication
ISCW008141	Salp26A, salivary secreted protein	Unknown [17]
ISCW013959	Putative secreted salivary protein	Tick salivary peptide group 1 [17]
ISCW024071	Secreted salivary gland protein	Unknown [17]
ISCW014240	5'Nucleotidase with a N-terminal metallophosphatase domain	Apyrase-like putative anticoagulant [17]

Supplementary Table 1. Upregulated genes in *Borrelia*-infected nymphal guts. List of protein-encoding genes preferentially increased in fed *B. burgdorferi*-infected *I. scapularis* guts identified using a subtractive hybridization approach. Only genes identified 5 or more times in the subtracted library screening are represented in the list. Asterisk represents genes identified 10 times in the library screening. Functional assignments are based on *I. scapularis* genome annotation (www.vectorbase.org) or based on BLAST analysis. Relevant published works related to the gene product or to their putative functions are referenced. PIXR is underlined.

Supplementary Table 2. Primers utilized in this study for quantitative RT-PCR assessment of gene expression profiles and for probe generation for *in situ* hybridization.

Gene Name	Forward primer sequence	Reverse primer sequence
Tick actin	ggcgacgtagcag	ggtatcgtgctcgactc
Mouse actin	agcgggaaatcgtgcgtg	cagggtacatggtggtgcc
flaB	ttcaatcaggtaacggcaca	gacgcrrgagaccctgaaag
pixr (KY629420)	tcacggatacattgccaaga	accgtgaccttgaagtggac*
pixr2 (KY865270)	gaggactacaagcc	tgtgcgtgatacc
pixr2FL	atgatggcactgaaggtagcaacgtcgctgatattg	ttttttgagcttttatagtctttatttagttctgttg
pixr3(ISCW018240)	gactacaagccgg	accgtgaccttgaagtggac*
pixr4(ISCW017732)	ggttcctgattgaggcc	aaccttgccggatctt
pixr5(ISCW005667)	tttcgacgagaacgagaagg	accgtgaccttgaagtggac*
pixr6 (ISCW000590)	cagttcgtgcagct	cagcgcttcgtaatt
pixr7(ISCW000233)	tgcctctcgctttaa	gggctgaatgtcctt
relish (ISCW018935)	accctctgctgcgtctactc	tcctcgtcctcctcaaagaa
bsk (ISCW020577)	cccgtaaggacccttctgtt	atttaaaacgcgcacaaacc
dorsal (ISCW000140)	gacgtgcaggtcctcttga	attcccctgaaagcttcctc
duox (ISCW007865)	tgcctctcgctttaa	acatgtacacgcgtttcagc
nos (ISCW018074)	acttcggactcccagctgta	atgacgtcgccacagacata
scapularisin1(ISCW005927)	cacaaccactgcagaagcat	tttaaattgccgaccgattc
scapularisin5(ISCW005926)	aagtggctcacgttcgagtt	ggtaacacgcctcctcacat
pgrp1(ISCW004389)	gggaaaatgcgcagaaatta	ttttggcacaagtttccaca
pgrp2 (ISCW024175)	ccaagttccgaatgagcgta	ccatgaaggcatggaagg
pgrp3(ISCW024689)	gctttggtgccattggag	tcaaagcgggacataccaattc
pgrp4 (ISCW022212)	tcacacccgaggatacaaca	agagaatgggtgctgctgat
16SUniversal	gtgccagcagccgcggtaa	ccgtcaatcMtttRagttt
Lysinibacillus 16S	cgcgagagggagctaatccgataaag	ctatcccaccttcggcggctggctccaa
Rickettsia 16S	cgtggagcaaatccctaaaa	cttgcgttagctcaccacct
Brevibacterium 16S	cgtctgctgtggaaacgcaa	ccttggtaaggttcttcg
Pseudomonas 16S	tgctgagaactttccagaga	gctaagggccatgatgactt
Acinetobacter 16S	tagagtatgggagagga	tttacggcatggactacc
Staphylococcus 16S	cggacgagaagcttgctt	gcatcgttgccttggta
Enterococcus 16S	cctgcccatcagaaggg	cctgtctcagtcccaatgtggccgatcacc
ospA	gttttgtaatttcaactgctgacc	ctgcagcttggaattcaggcactt
bba74	actattaacaaggtaaccgaagatg	ccttgtttgcaccctcagcaac
rpoS	cttgcaggacaaatacaaagaggc	gcagctcttattaatcccaattgcc
bba62	tgagatggaaaatactagcgatga	tggtatttttgtctggcat
bb0690	ggaaagcattgtttgcagtctta	agtaccataatcaccagcagtatc
bbb29	atctgcccttaatgtttgcagcgg	agcttgcgctttctggtgtgattc

ESI-Positive Mode	ESI-Negative Mode
1-Stearoyl-sn-glycerol	2,3-Dihydroxybenzoic acid
2'-Deoxyadenosine 5'-monophosphate (dAMP)	2'-Deoxyadenosine 5'-monophosphate (dAMP)
3'-O-methylguanosine	2'-Deoxyguanosine 5'-monophosphate (dGMP)
4-Guanidinobutyric acid	2'-O-methyladenosine
5-L-Glutamyl-L-alanine	2-Oxoadipic acid
Acetylcarnitine	3-Hydroxy-L-kynurenine
Adenine	3-Hydroxypropionic acid (beta-lactic acid)
Adenosine	5-Hydroxyindoleacetate
alpha-D-Glucose 1-phosphate	5-L-Glutamyl-L-alanine
Argininosuccinic acid	6-Phospho-D-gluconate
Betaine	Acetyl phosphate
Biliverdin	Acetylglycine
Creatine	Adenine
Cytidine	Adenosine
Cytidine 5'-diphosphocholine (CDP-choline)	Arachidonic Acid (peroxide free)
D-Alanyl-D-alanine (D-Ala-D-Ala)	cis-9-Palmitoleic acid
Dimethylglycine	cis-Aconitate
Dopamine	Citrate
Glycerophosphocholine	Cytidine 5'-diphosphocholine (CDP-choline)
Guanosine	Cytidine 5'-monophosphate (CMP)
Hypoxanthine	D-Glucosamine 1-phosphate (Glucosamine-1P)
Indole-3-pyruvic acid	Dihydrouracil
Inosine	D-Maltose
L-Arginine	D-Ribose
L-Asparagine	Eicosapentaenoic acid
L-Citrulline	gamma-L-Glutamyl-L-valine
L-Histidine	Glutathione disulfide
L-Kynurenine	Glyceraldehyde 3-phosphate
L-Methionine	Glycerol 3-phosphate
L-Phenylalanine	Glycerophosphocholine
L-Pipecolic acid	Glycolate
L-Pyroglutamic acid	Glyoxylate
L-Saccharopine	Guanosine 5'-monophosphate (GMP)
L-Tryptophan	Guanosine diphosphate mannose
Maltotriose	Indole
N-(omega)-Hydroxyarginine	Inosine
N,N'-Diacetylchitobiose	Inosine 5'-monophosphate (IMP)

N6,N6,N6-Trimethyl-L-lysine	L-Arginine
N6-Acetyl-L-lysine	L-Glutamate
N-Acetyl-D-Glucosamine 6-Phosphate	L-Histidine
N-Benzyloxycarbonylglycine	Linoleic acid
NG,NG-dimethyl-L-arginine(ADMA)	L-Methionine
Nicotinamide	L-Phenylalanine
Nicotinate	L-Pyroglutamic acid
Oxypurinol	L-Tryptophan
PC(16:0/16:0)	Maltotriose
Pristanic acid	Methylmalonic acid
Protoporphyrin IX	N-Acetyl-D-Glucosamine 6-Phosphate
Pyridoxal (Vitamin B6)	N-Acetylglucosamine 1-phosphate
S-Adenosyl-L-homocysteine	N-Acetylneuraminic acid
S-Adenosylmethionine	Palmitic acid
S-Methyl-5'-thioadenosine	Phosphoenolpyruvate
sn-Glycerol 3-phosphoethanolamine	Pyridoxamine 5'-phosphate
Spermidine	Quinolinate
Taurine	S-Adenosyl-L-homocysteine
UDP-N-acetylglucosamine	sn-Glycerol 3-phosphoethanolamine
Uridine 5'-diphosphate (UDP)	Taurine
Uridine 5'-monophosphate (UMP)	Thymine
Xanthine	UDP-N-acetylglucosamine
	Uracil
	Uridine 5'-monophosphate (UMP)
	Uridine diphosphate glucose(UDP-D-Glucose)
	Urocanic acid
	Xanthosine

Supplementary Table 3. List of metabolites identified by Ultra performance liquid chromatography–tandem mass spectroscopic analysis of tick guts. ESI, Electrospray ionization mass spectrometry.

**Bacterial Genera**	**Buffer**	**Larvae (Ova/PIXR)**
Acinetobacter	4.0	35-45
Pseudomonas	~1	8-36
Brevibacterium	~1	4-20
Lysinibacillus	<1	0.76-5.8
Enterococcus	0.5	3.2
Staphylococcus	0.5	2-4
Rickettsia	-	11-16
*Chryseobacterium**	0.5	1.3
*Stenotrophomonas**	<1	35-70
Klebsiella	2.4	2-2.5
Corynebacterium	6.2	0.7-3.5
Proteus	7.0	1

Supplementary Table 4. Predominant bacterial genera in larval ticks. Relative percent abundance of predominant bacterial genera in larval ticks fed on Ovalbumin or PIXR-immunized mice and in control/buffer samples. 16S rRNA corresponding to genera marked by asterisk could not be consistently amplified in tick samples by qRT-PCR. Bacterial Genera present at =/> abundance in mock sample are underlined.

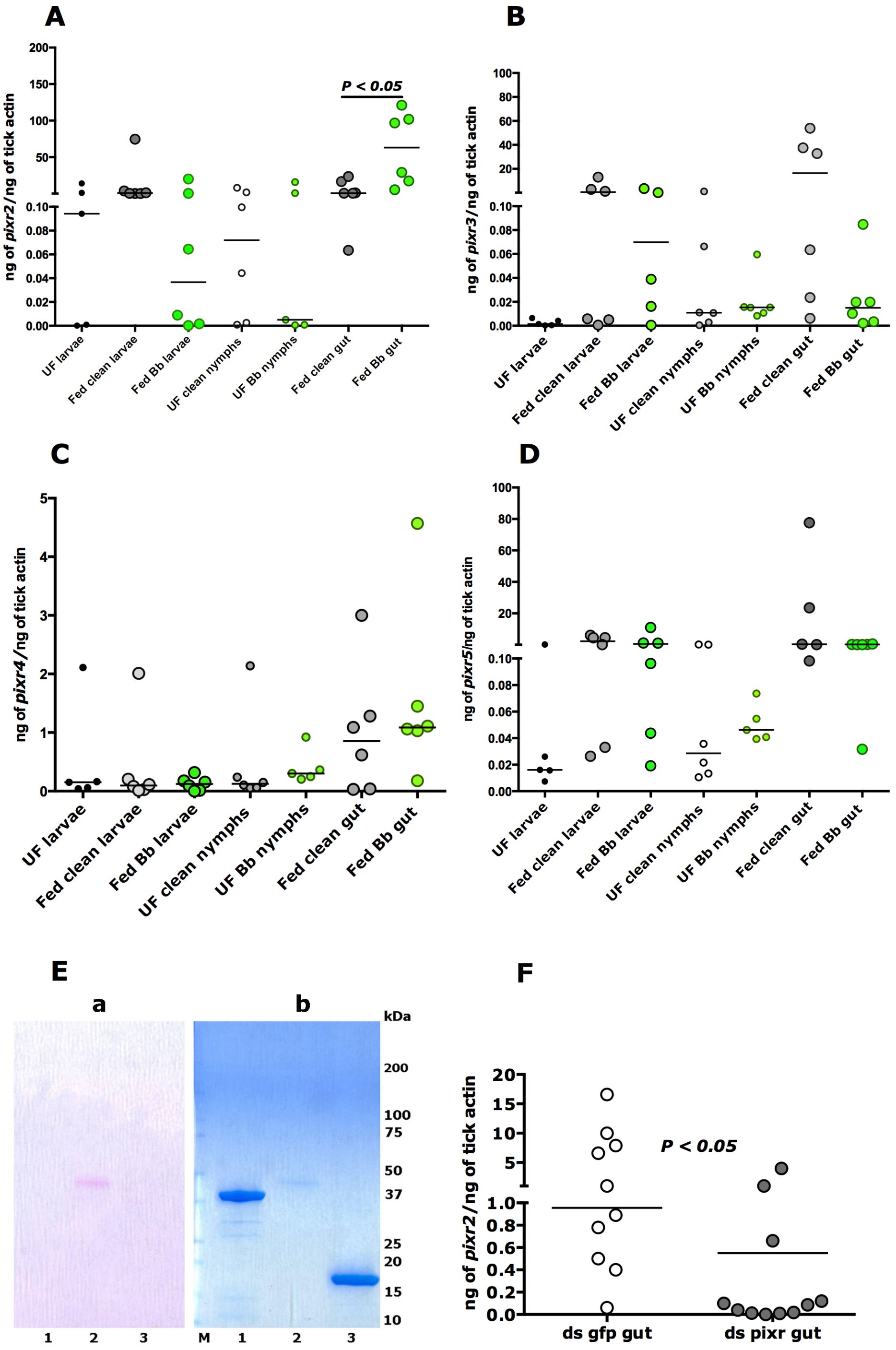

Supplementary Figure 1. **Expression profile of *pixr* paralogs**. qRT-PCR assessment of the transcript levels of *pixr2* (**A**); *pixr3* (**B**); *pixr4* (**C**); and *pixr5* (**D**) in unfed larvae (UF larvae), in larvae fed on pathogen-free (Fed clean larvae) or *B. burgdorferi*-infected (Fed Bb larvae) mice, in unfed pathogen-free (UF clean nymphs), unfed *B. burgdoferi*-infected (UF Bb nymphs) nymphs, fed pathogen-free (fed clean gut) and fed *B. burgdorferi*-infected nymphal guts (Fed Bb gut). Each data point refers to a pool ~50 unfed larvae, 5 fed larvae, 5 unfed nymphs, or 2-3 fed nymphal guts. **E**.**a**. Gycostain and **b**. Coomassie stain of: Lane1, rGlpQ; lane 2, Horse radish peroxidase; lane 3, rPIXR. **F**. qRT-PCR assessment of *pixr2* transcript levels in the guts of ds *gfp* or ds *pixr* RNA-injected nymphs fed on mice. Each data point represents a pool of 2-3 guts. Horizontal bars represent medians. Mean values significantly different in a non-parametric Mann-Whitney test ($P < 0.05$) indicated.

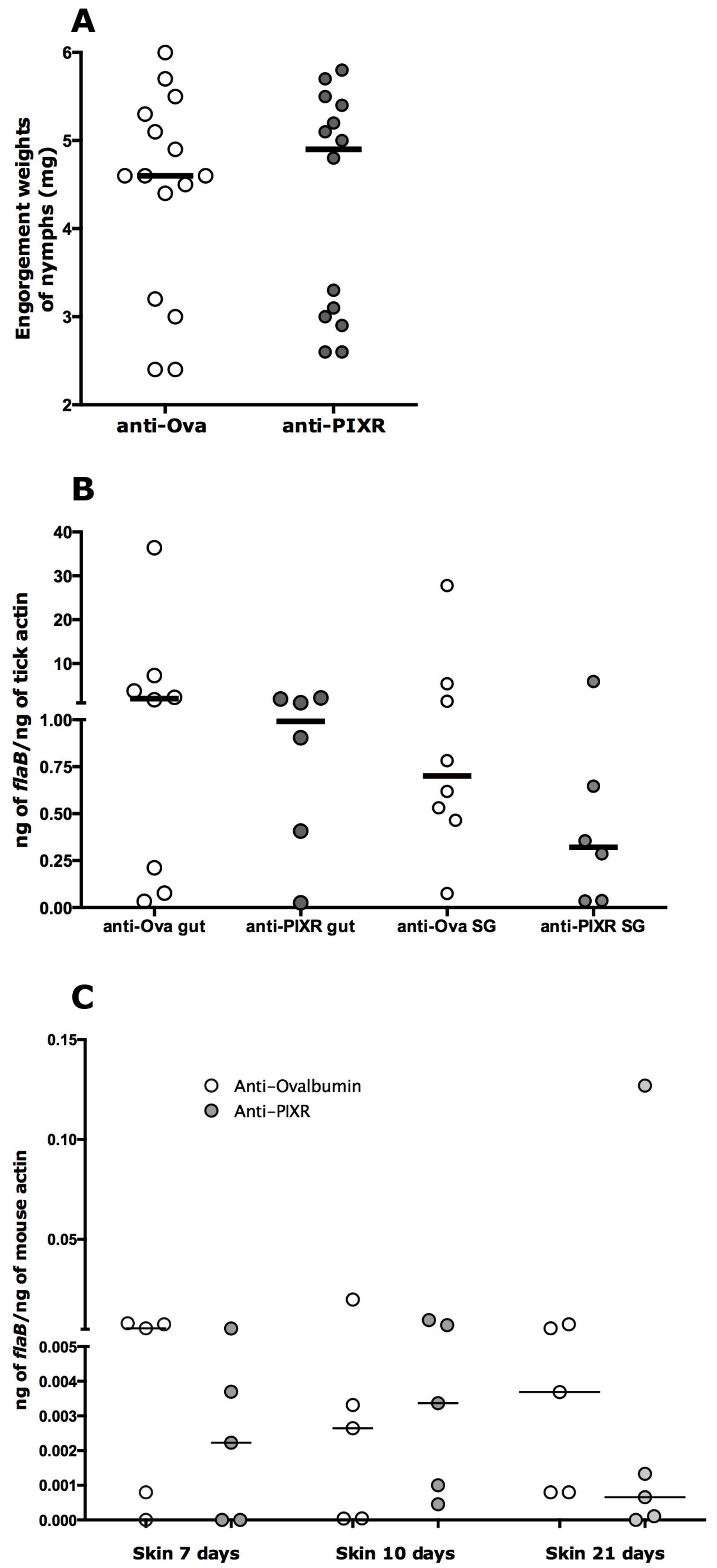

Supplementary Figure 2. PIXR abrogation does not influence *B. burgdorferi* transmission to the murine host. *B. burgdorferi*-infected nymphs were fed on Ovalbumin-(anti-Ova) or rPIXR (anti-PIXR)-immunized mice: **A**. Nymphal engorgement weights; **B**. qRT-PCR assessment of *B. burgdorferi flaB* levels in nymphal guts (Guts) and salivary glands (SG); **C**. qPCR assessment of *B. burgdorferi flaB* levels in the skin of mice at 7, 10, and 21 days-post tick repletion. Horizontal bars represent the median.

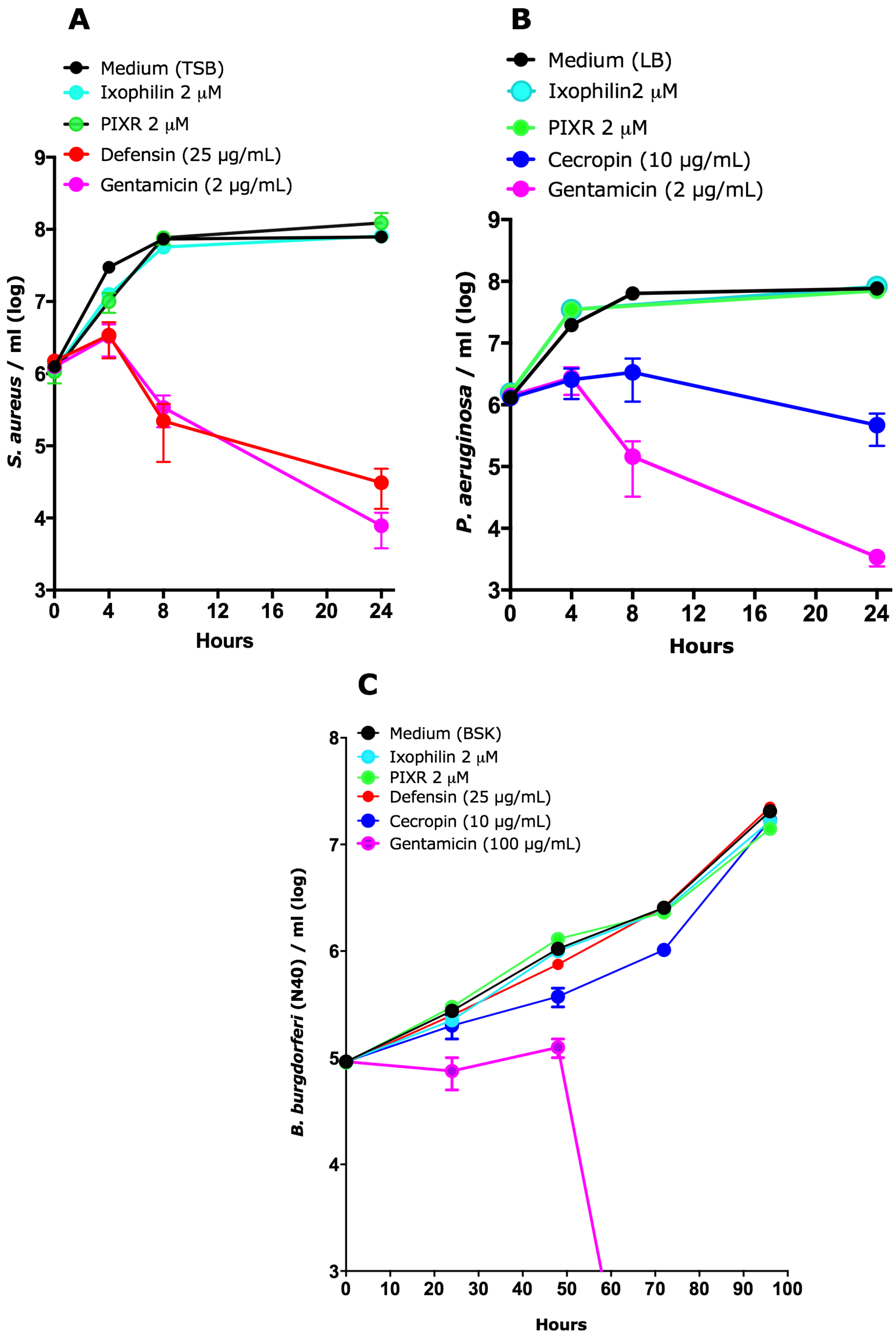

Supplementary Figure 3. PIXR is not bactericidal. Growth of: **A.** *Staphylococcus aureus*; **B.** *Pseudomonas aeruginosa*; and **C.** *B. burgdorferi* N40, were assessed for a maximum of 24 (for *S. aureus* and *P. aeruginosa*) and 96 hours (for *B. burgdorferi* N40) in the presence of recombinant PIXR (rPIXR), Ixophilin (rIxophilin), respective medium, Cecropin, or Defensin. Data are means ± SEM of at least 3 replicates and representative of triplicate experiments

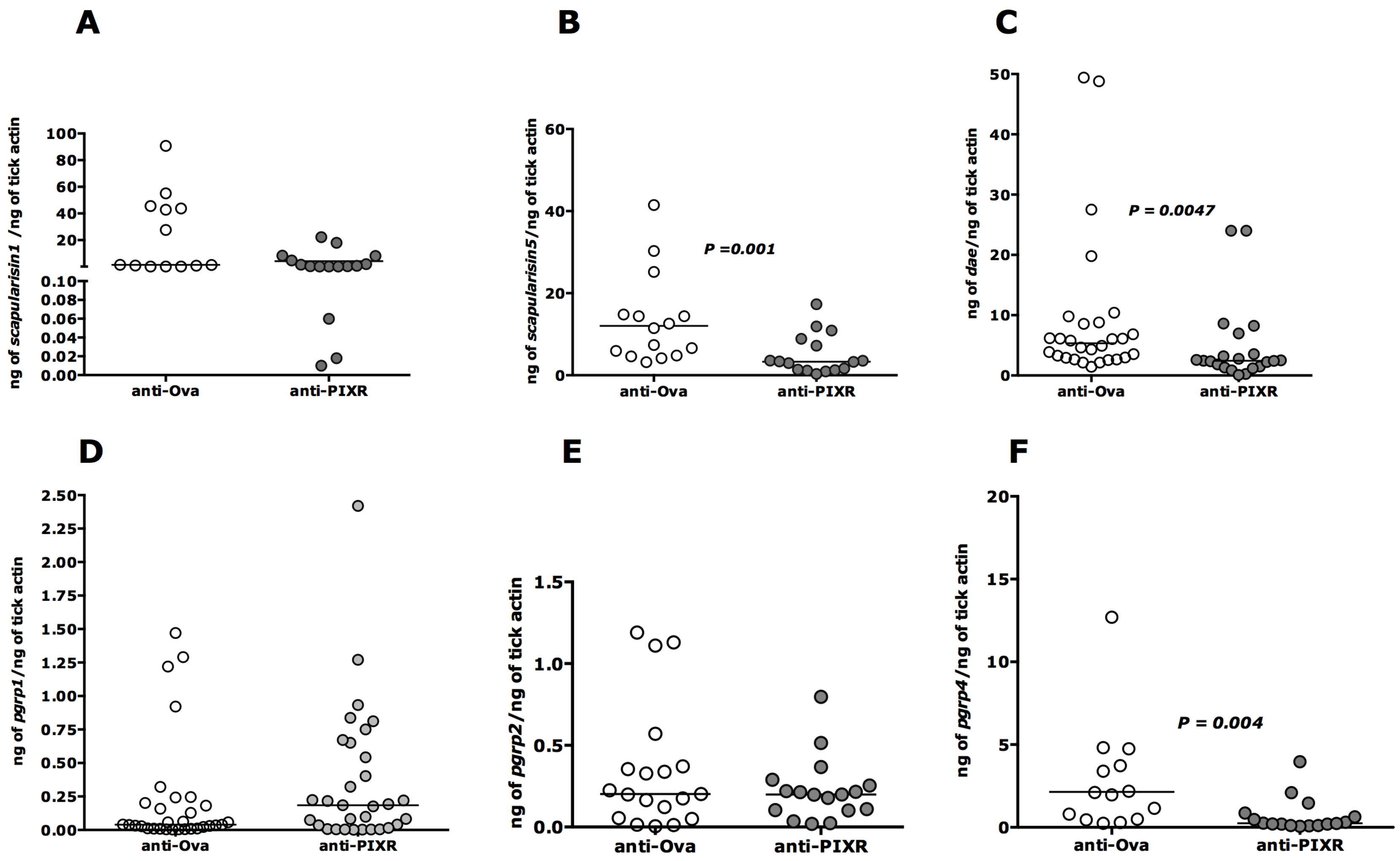

Supplementary Figure 4. **PIXR abrogation and impact on tick immune response genes.** qRT-PCR analysis of expression levels of: **A**. *scapularisin1;* **B.** *scapularisin5;* **C**. *dae;* **D**. *pgrp1,* **E**. *pgrp2* and **F**. *pgrp4* in repleted larvae fed on rPIXR-immunized or Ovalbumin-immunized *B. burgdorferi*-infected mice. Each data point represents a pool of 5 ticks. Horizontal bars represent the median. Mean values significantly different in a non-parametric Mann-Whitney test ($P < 0.05$) indicated.

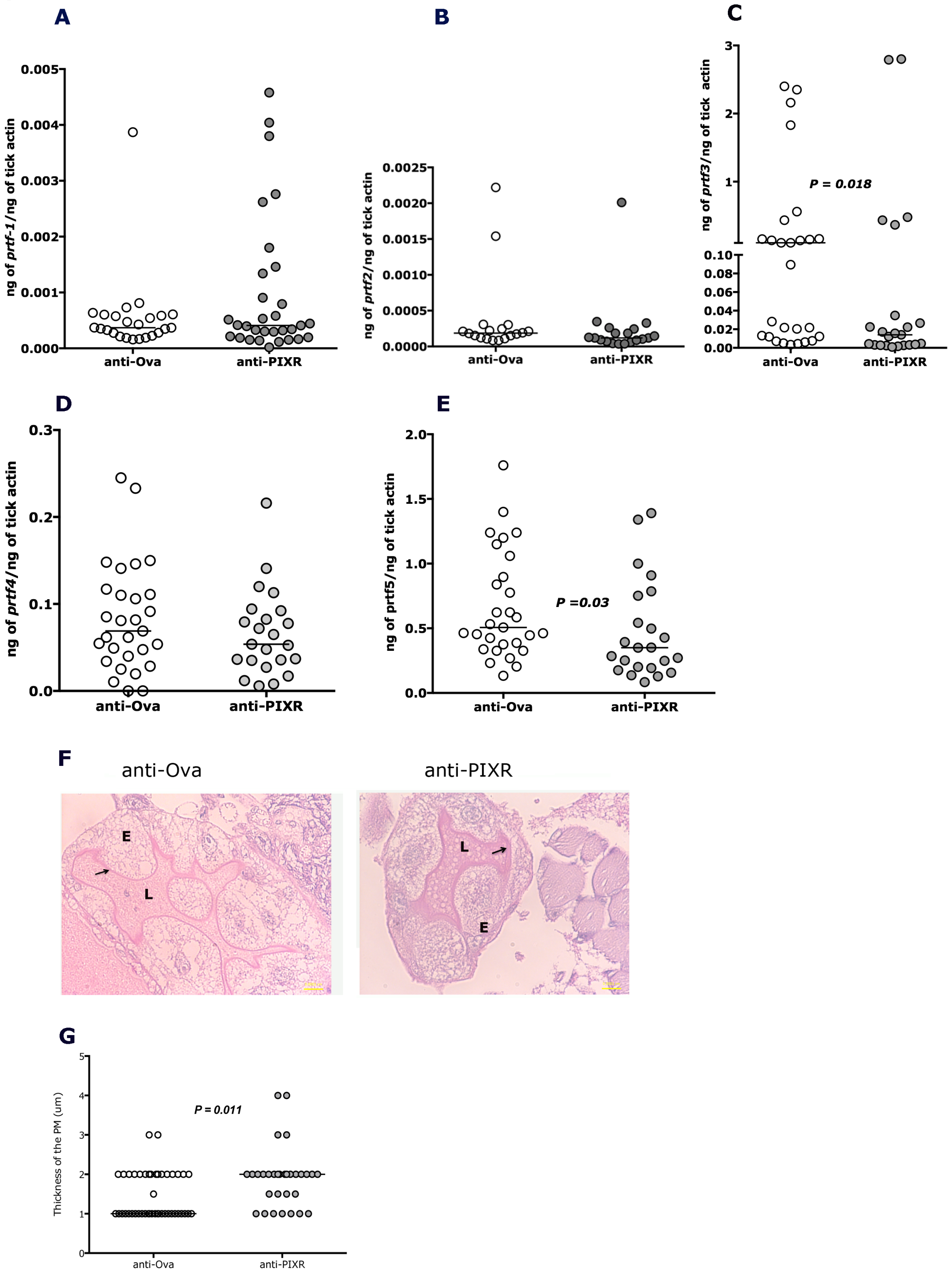

Supplementary Figure 5. PIXR abrogation and impact on the expression of *peritrophin transcripts* and on the peritrophic matrix. qRT-PCR analysis of expression levels of *peritrophins* (*prtf*): **A**. *prtf1;* **B.** *prtf2;* **C**. *prtf3,* **D**. *prtf4* and **E**. *prtf5* in repleted larvae fed on rPIXR-immunized or Ovalbumin-immunized *B. burgdorferi*-infected mice. Each data point represents a pool of 5 ticks. Horizontal bars represent the median. Mean values significantly different in a non-parametric Mann-Whitney test ($P < 0.05$) indicated. **F**. Periodic acid Schiff's stain of the peritrophic matrix (PM, indicated by an arrow) of nymphs fed on rPIXR-immunized (anti-PIXR) or Ovalbumin-immunized (anti-Ova) *B. burgdorferi*-infected mice for 48 h. Epithelium (E) and lumen (L); **G**. quantitative assessment of the PM. Mean values significantly different in a non-parametric Mann-Whitney test ($P < 0.05$) indicated.

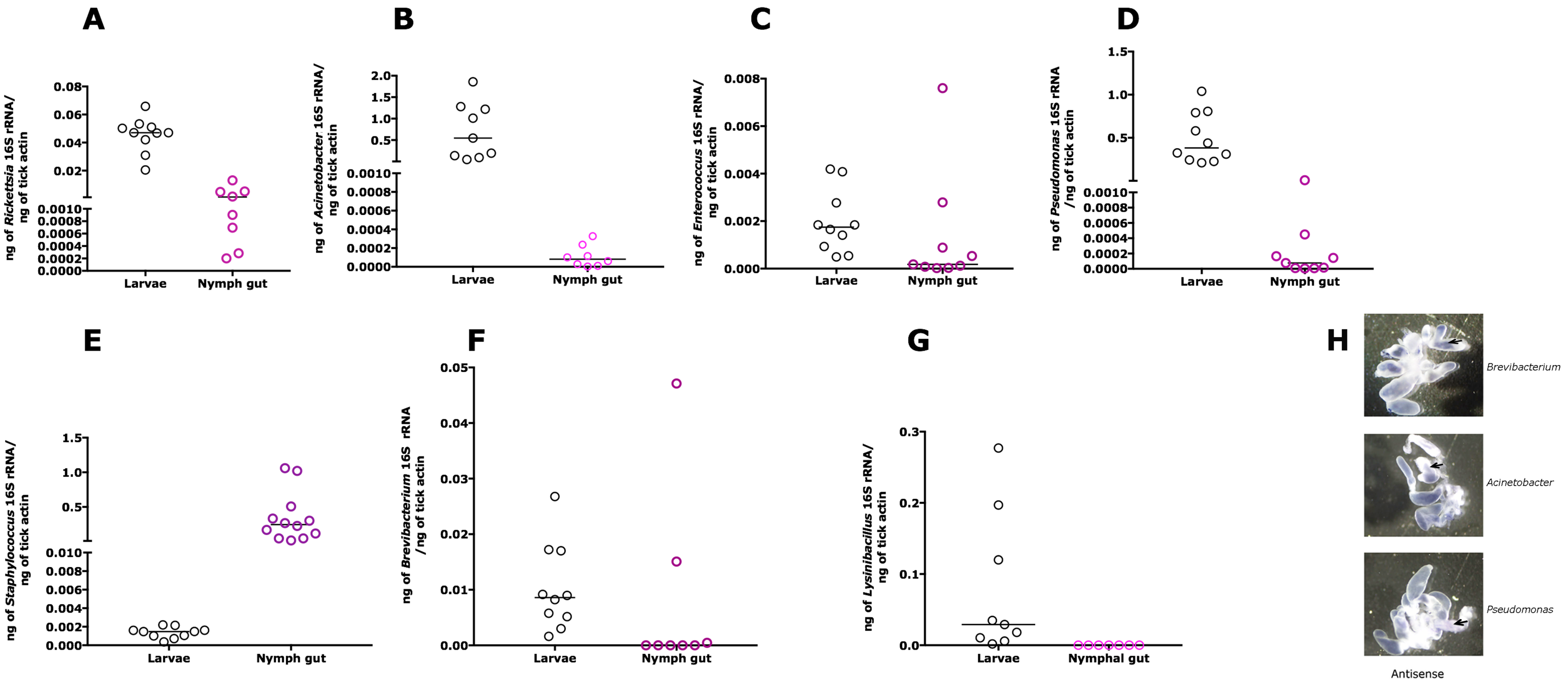

Supplementary Figure 6. Representation of bacterial genera in larval and nymphal ticks. qRT-PCR assessment of genera-specific 16S rRNA transcripts in the guts of fed nymphs and repleted larvae. **A.** *Rickettsia. B. Acinetobacter*. **C**. *Enterococcus*. **D**. *Pseudomonas*. **E**. *Staphylococcus*. **F**. *Brevibacterium* and **G**. *Lysinibacillus*. Each data point represents a pool of 5 larval ticks or 2-3 nymphal guts. **H**. Whole-mount *in situ hybridization* of antisense genera-specific 16S rRNA probes in 48 h-fed guts of nymphs fed on pathogen-free mice. Arrows indicate specific staining.

A

```
pixr    ATGT---------------------CGATGGTCTTGGTCTTCTTGTTTGC
pixr2   ATGATGGCACTGAAGGTAGCAACGTCGCTGATATTGGTCCTCCTGTCTGC
        ***                      ** ** * ****** ** *** ***

pixr    TGGAATCTGCGTGGGACACCCGGACGGAGCTGACGACGAGGCTTGCGCAC
pixr2   TGTAATTTGCACGGGACATCCTGACGGAGCTGACGACGAGGCCTGCAAAG
        ** *** ***  ****** ** ******************** ***  * 

pixr    ACCTTTTTCCGTATCACGGATACATTGCCAAGAGCGCCGCTGAGGGTCGC
pixr2   ACCTGTATCCGTCCCACGGATACACTGCCAAGAGCGCTGCTGAGGGCCAC
        **** * *****  ********** ************ ******** * *

pixr    GCCGAGGGCTACCGTCTCGTCCAAAACAAAAAAGACTACAAGCCCGGCGA
pixr2   GCCGAGGGCTACCGTCTCGTCCAAGACACAGAGGACTACAAGCCGGGTGA
        ************************ *** * * *********** ** **

pixr    CGTCATCACAGTGACGCTGTCCTCGGAGGCTTCTCCTTTCATGGGGTTCC
pixr2   CGTCATCACAGTGACACTGTCCTCGGAGGCCTCTCCCTTCATGGGGTTCC
        *************** ************** ***** *************

pixr    TCATCAAGGCCTTCGACGAGGACGAGATGGACGTGGGCTCCTTCAGGAGT
pixr2   TGATCAAGGCTTTCGACGAGAACGAGAAGGATGTGGGCTCCTTCAGGAGC
        * ******** ********* ****** *** ***************** 

pixr    ACCGGACCGGACTCGAGGGCATTCAGCCACTGCTCTGGAATCACGCACAC
pixr2   ACCGGTCCCGACTCGAGGGCATTCAGCCACTGCGCCGGTATCACGCACAC
        ***** ** ************************ * ** ***********

pixr    CTCGAAGAACCTGAAGAAGAGGGTCGTGGTGCAGTGGCTGGCTCCAGAGG
pixr2   CTGGAGGGACCTGAAGAAGAGGGTTGTCGTCCAGTGGATGGCACCAGAGG
        ** ** * **************** ** ** ****** **** *******

pixr    ACAGGTCGGGTGAGGTCCACTTCAAGGTCACGGTCGTCAAAGAATTCAAG
pixr2   ACAGATCCGGCAAGGTCCACTTTAAGGTCACAGTCGTCAAACAAGTAGAG
        **** ** **  ********** ******** ********* ** *  **

pixr    GACTTCTACCACGCCATTCCAAGCACGTTGGCTTAA
pixr2   GACTTCTACCACGCCATTCCAAGCACGTTGGCTTAA
        ************************************
```

B

```
PIXR    M-------SMVLVFLFAGICVGHPDGADDEACAHLFPYHGYIAKSAAEGR
PIXR2   MMALKVATSLILVLLSAVICTGHPDGADDEACKDLYPSHGYTAKSAAEGH
        *       *::**:* * **.*********** .*:* *** *******:

PIXR    AEGYRLVQNKKDYKPGDVITVTLSSEASPFMGFLIKAFDEDEMDVGSFRS
PIXR2   AEGYRLVQDTEDYKPGDVITVTLSSEASPFMGFLIKAFDENEKDVGSFRS
        ********:.:*****************************:* *******

PIXR    TGPDSRAFSHCSGITHTSKNLKKRVVVQWLAPEDRSGEVHFKVTVVKEFK
PIXR2   TGPDSRAFSHCAGITHTWRDLKKRVVVQWMAPEDRSGKVHFKVTVVKQVE
        ***********:***** ::*********:*******:*********:.:

PIXR    DFYHAIPSTLA
PIXR2   DFYHAIPSTLA
        ***********
```

Supplementary Figure 7. **Alignment of the nucleotide and protein sequences of *pixr* and *pixr2*.** **A**. The full-length nucleotide sequences of *pixr* and *pixr2* were aligned using the T-Coffee multiple sequence alignment program (tcoffee.crg.cat). Asterisks represent identities, dashes indicate gaps. The ds pixr RNA region is boxed and the black overlines within the box indicate the primers used to generate ds pixr. **A**. The full-length protein sequences of PIXR and PIXR2 were aligned using the T-Coffee multiple sequence alignment program (tcoffee.crg.cat). Identities, conservative and non-conservative changes indicated as (*), (:) and (.) respectively.

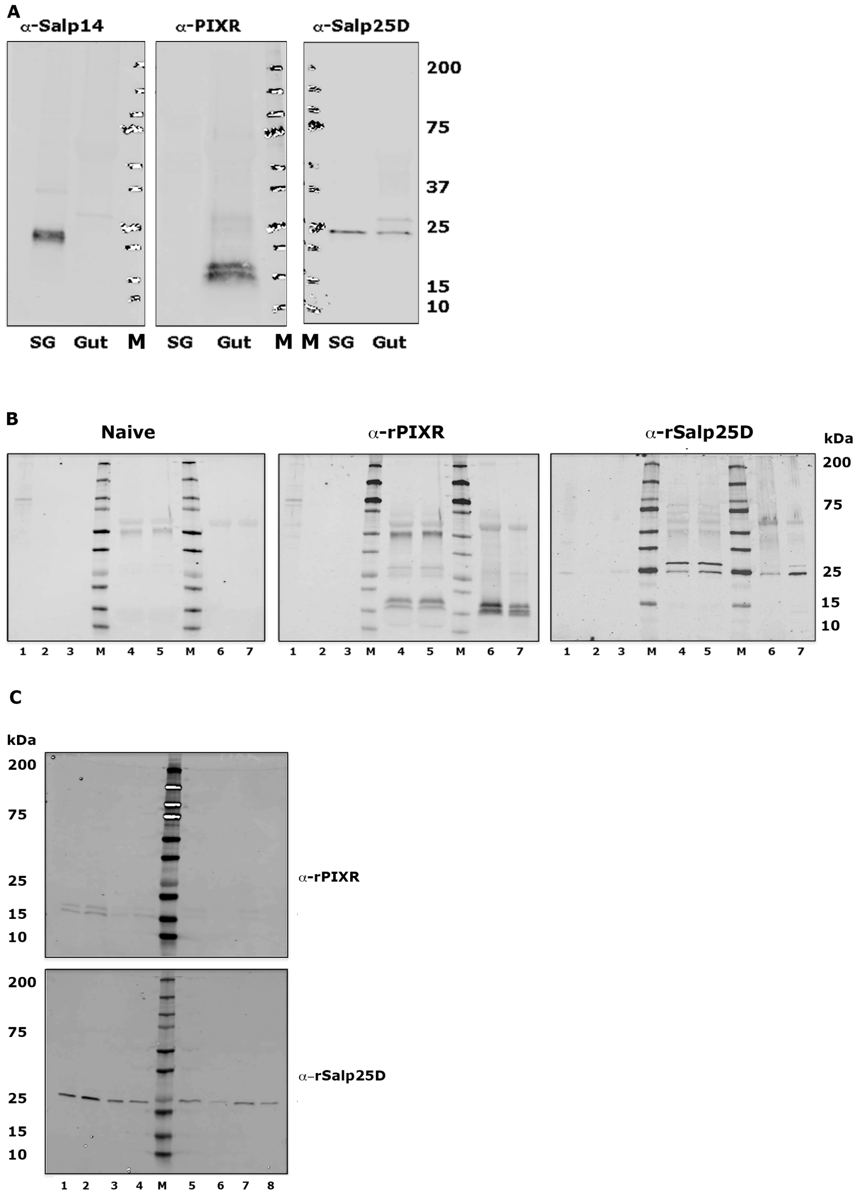

Supplementary Figure 8. **Full-length western blot images to assess PIXR expression**. **A**. western blot assessment of PIXR expression in comparison with Salp14 and Salp25D expression in fed nymphal salivary glands (SG) and guts (Gut) using polyclonal rabbit ati-recombinant Salp14 (α-rSalp14) or anti-recombinant PIXR antibodies (α-rPIXR) or anti-recombinant Salp25D (α-rSalp25D). Naïve rabbit serum (Naïve) was used to probe a replicate blot. Replicate blots are boxed and protein markers (Precision Plus Biorad protein markers) are shown in lane M and their cognate molecular weights denoted to the right. Markers appear white due to saturation of signal. **B**. Replicate western blots of protein extracts of unfed larvae lane1; unfed *B. burgdorferi*-infected nymphal gut, lane 2; unfed clean nymphal gut, lane 3; larvae fed on *B. burgdorferi*-infected, lane 4; or on clean mice, lane 5; guts of fed *B. burgdorferi*-infected nymphs lane 6; or, fed clean nymphs lane 7; probed with polyclonal naïve rabbit sera, or polyclonal anti rPIXR or anti rSalp25D rabbit sera. Protein markers (Precision Plus Biorad protein markers) are shown in lane M and their cognate molecular weights denoted to the left. **C**. Western blot assessment of PIXR and Salp25D expression using a pool of 2 guts/lane. Lanes 1-4, ds *gfp* RNA-injected ticks and lanes 5-8, ds *pixr* RNA-injected ticks; Protein markers (Precision Plus Biorad protein markers) are shown in lane M and their molecular weights denoted to the left.
